# Complexity of social media in the era of generative AI

**DOI:** 10.1093/nsr/nwae323

**Published:** 2024-09-19

**Authors:** Liming Pan, Chong-Yang Wang, Fang Zhou, Linyuan Lü

**Affiliations:** School of Cyber Science and Technology, University of Science and Technology of China, China; Institute of Fundamental and Frontier Studies and Yangtze Delta Region Institute (Huzhou), University of Electronic Science and Technology of China, China; Institute of Fundamental and Frontier Studies and Yangtze Delta Region Institute (Huzhou), University of Electronic Science and Technology of China, China; School of Cyber Science and Technology, University of Science and Technology of China, China; Institute of Fundamental and Frontier Studies and Yangtze Delta Region Institute (Huzhou), University of Electronic Science and Technology of China, China

## Abstract

The article presents a perspective on how the emerging generative AI technology can shape social media and the new challenges in studying social media in this generative.

In recent years, we have witnessed a profound expansion of generative artificial intelligence (GenAI) creativity, driven by breakthroughs such as language generation and image generation. Large language models, exemplified by ChatGPT [1], now excel at creating vast quantities of natural-sounding text, and text-to-image models like Stable Diffusion [2] can produce remarkably realistic images.

The rapid advancement of GenAI has sparked extensive discussions regarding its impact on the social media landscape. On the positive side, GenAI offers the potential to swiftly create a large volume of captivating and realistic content tailored to individual preferences and deliver personalized recommendations, which previously demanded significant time and human efforts. Furthermore, GenAI can aid in identifying and filtering out harmful contents on social media platforms. However, these advantages come hand in hand with risks. AI algorithms may inadvertently generate false information without adequate training and supervision. Additionally, social robots equipped with GenAI could potentially wield significant influence while posing challenges in detection, thereby facilitating covert social media manipulations.

The influence of GenAI on social media goes beyond the examples provided. Constructing a model for online social networks integrated with GenAI is essential to gaining a more comprehensive understanding. In fact, over the past two decades, researchers have extensively studied information propagation from the perspective of complex networks and assumed that, as a complex system, a social media network is primarily self-organizing without or with scarce central management. Today, using GenAI and AI bots in social media can result in a more severe social media manipulation, potentially leading to a more centralized or ‘synthetic’ social media. In this sense, GenAI can introduce drastic changes in social media network structures and information propagation mechanisms.

To better understand the underlying mechanism of how GenAI changes the social media network structure and spreading dynamics by influencing individual users, this paper first discusses information propagation modeling within GenAI-involved complex networks. Subsequently, we delve into the identification of artificial intelligence generative content (AIGC) and social bots within these networks. Finally, we explore how GenAI influences network intervention practices. Figure 1 shows the organization and main points of the paper. This paper presents a perspective on modeling the intricate process of information propagation on social media in the era of GenAI. It aims to provide understanding for addressing misinformation, combating social media manipulation and enabling effective regulation of GenAI technologies in this era. Such efforts will be instrumental in fostering a responsible and ethical integration of artificial intelligence into our society.

**Figure 1.**
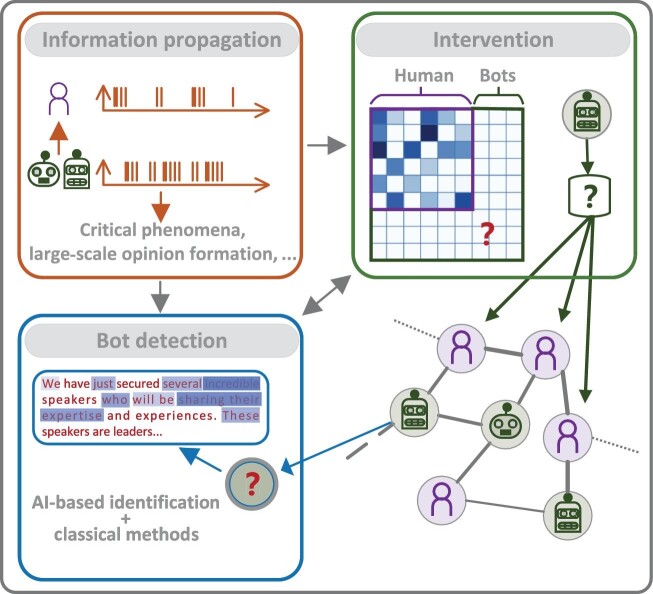
An illustration of three main challenges and their connections. In order to model information propagation, the first step is to characterize the AI bots in their temporal active patterns, spatial distributions, how they impact a human user and so on. With this knowledge, we can build models to understand macroscopic behaviors of social media, such as critical phenomena and large-scale opinion formation. These features also provide a basis for bot detection, which AI-based identification techniques can further aid. Information propagation and bot identification then build the basis for network intervention, e.g. to remove or distribute bots optimally.

## INFORMATION PROPAGATION MODELING

Understanding the underlying information-spreading mechanism at the microscopic individual level is necessary to accurately characterize the potentially new collective behaviors emerging from online social networks in the GenAI era. The information propagation mechanism for social media is generally determined by where and when content will be created, shared and consumed. Specifically, it depends on factors such as users’ interests, temporal activation patterns and connection patterns. With the introduction of GenAI techniques in social media, all of these factors may undergo changes; see the top-left panel in Fig. 1 for an illustration. For example, a GenAI agent can generate high-quality and creative content tailored to a human user’s interests, which are, on average, more likely to be shared. In addition, a GenAI agent can have distinct temporal activation patterns compared to a human user, where temporal factors like weekdays, holidays or the time of day that are important for human users might become irrelevant. Furthermore, with the aid of big data analysis, a GenAI user is prone to showcasing a more diverse array of connections. In contrast, connections formed by a human user typically lean towards homogeneity, shaped by personal relationships and individual interests.

All of the aforementioned individual-level changes in information spreading may affect the collective behaviors at the macroscopic level, such as the propagation outbreak threshold, public opinion formation and the emergence of echo chamber effects. If intentionally designed, GenAI agents have the capability to affect information spreading on a larger scale and exert profound influence. For instance, a GenAI agent with extensive connections in online social networks could be employed to spread a significant quantity of false political news, misguide public opinions and potentially manipulate an election. Apart from influencing the dynamics of information distribution, GenAI can also shape the topology of social media networks. For instance, a large number of AI bots can be deployed to interact with other users, leading to the emergence of new super-spreaders and communities. Studies have also shown that GenAI agents can self-organize to form network structures with particular topological properties [3].

The rise of AI technology has brought many changes to social media networks, potentially rendering the classical paradigm of mechanical modeling less effective. Yet it also blesses us with a new tool for studying complex systems known as scientific machine learning. These various AI algorithms provide powerful tools for data analysis, pattern recognition and prediction, which can be helpful for information-spreading-mechanism modeling [4], network structure inference [5] and future state forecast [6] in complex networks. Because of social media’s intricacy, the spreading mechanisms are typically not fully known. AI methods can reduce noise and irrelevant factors in data, learn interaction rules and build surrogate dynamical models at multiple scales for forecasting system evolution. It is expected that an algorithmic toolbox of consistent data-driven social media modeling will aid in addressing the complexity of social media systems in the era of GenAI.

## SOCIAL BOT DETECTION

The concept of social bots dates back to the early period of computers, and their detection has long been a subject of interest due to their potential harm, such as amplifying the spread of misinformation [7]. Classical detection methods can be categorized [8] into graph-based, feature-based and crowdsourcing approaches. Graph-based methods assume that social bots have different connectivity patterns compared to human users and identify anomalies indicative of bot activity by analyzing network structures. Feature-based methods focus on users’ behavioral patterns, encoding them into features that are then used with machine learning techniques to learn the signatures of human-like and bot-like behaviors. In contrast, crowdsourcing methods leverage human intelligence to identify social bots, involving a substantial number of human workers in the detection process. Accurately characterizing their connectivity patterns and features is required to generalize these classical methods to the new GenAI bots.

Today, armed with AI techniques, social bots increasingly resemble human users in their connection patterns and behavioral traits. This similarity makes it more challenging to distinguish them from human users and effectively detect their presence. In response to this challenge, one approach is to tailor classical methods to the new features of GenAI bots, while another involves leveraging AI techniques for detection; see the bottom-left panel of Fig. 1. The emergent area of AI security has been focusing on techniques to make AIGC recognizable. For example, SynthID [9] watermarks the text, image or audio and detects AIGC by identifying the watermarks. GPTZero [10] uses qualities called perplexity and burstiness to attempt to determine if an AI wrote a passage. As GenAI bots advance, it is crucial to ensure that our detection technologies keep pace with them. Identifying social bots will significantly enhance our understanding of their distinctive characteristics, which will, in turn, assist in modeling information propagation within social networks in the era of GenAI.

## NETWORK INTERVENTIONS

Developing a robust model that integrates GenAI bots can significantly enhance the effectiveness of network intervention strategies, which fundamentally revolve around optimizing the dynamic propagation mechanisms. Specifically, network interventions harness social network data to accelerate behavioral change and enhance organizational performance [11], including tasks such as influence maximization and addressing pressing challenges like polarization and the echo-chamber effect within networks. These processes can be regarded as the inverse optimization of network propagation (see the bottom-right panel of Fig. 1), highlighting the necessity of an accurate dynamical model that integrates GenAI bots. Such a model is the basis for any further intervention designs. With the continuous advancement of AI techniques, traditional intervention approaches must evolve to accommodate the unique characteristics of GenAI bots and their generated content.

When formulating intervention strategies for GenAI-involved networks, it is crucial to recognize the substantial differences between GenAI bots and human users so as to tailor strategies effectively to suit these distinctions. In addition, a more profound change that demands attention is the scale of possible interventions. In particular, classical intervention practices for online social networks are typically small scale and lead to minor perturbations in network dynamics. However, since the GenAI bots are scalable in their amounts within current online social networks, the intervention could be far beyond ‘perturbative’ and requires global optimal design. This means that the collective effect is much more significant, making combinatorial optimization over dynamical processes even harder.

## CONCLUSIONS

The generative AI technique is still rapidly evolving. How this emerging technology will shape social media and, eventually, human society remains uncertain. Systematic studies on information propagation mechanisms, social bot detection and network interventions within GenAI-involved networks will offer insights into this inquiry. Yet, while the AI technique presents numerous challenges to modeling social media networks, it also offers effective tools to aid in constructing these models. Studies of social media in the era of GenAI require a long-term interdisciplinary effort from AI and network science and other related fields.
